# Supplementary material for: Specific N-cadherin–dependent pathways drive human breast cancer dormancy in bone marrow
Source: Life Sci Alliance. 2021 Jun 2;4(7):e202000969. doi: 10.26508/lsa.202000969 (PMC8200294; doi:10.26508/lsa.202000969)

Figure 1A

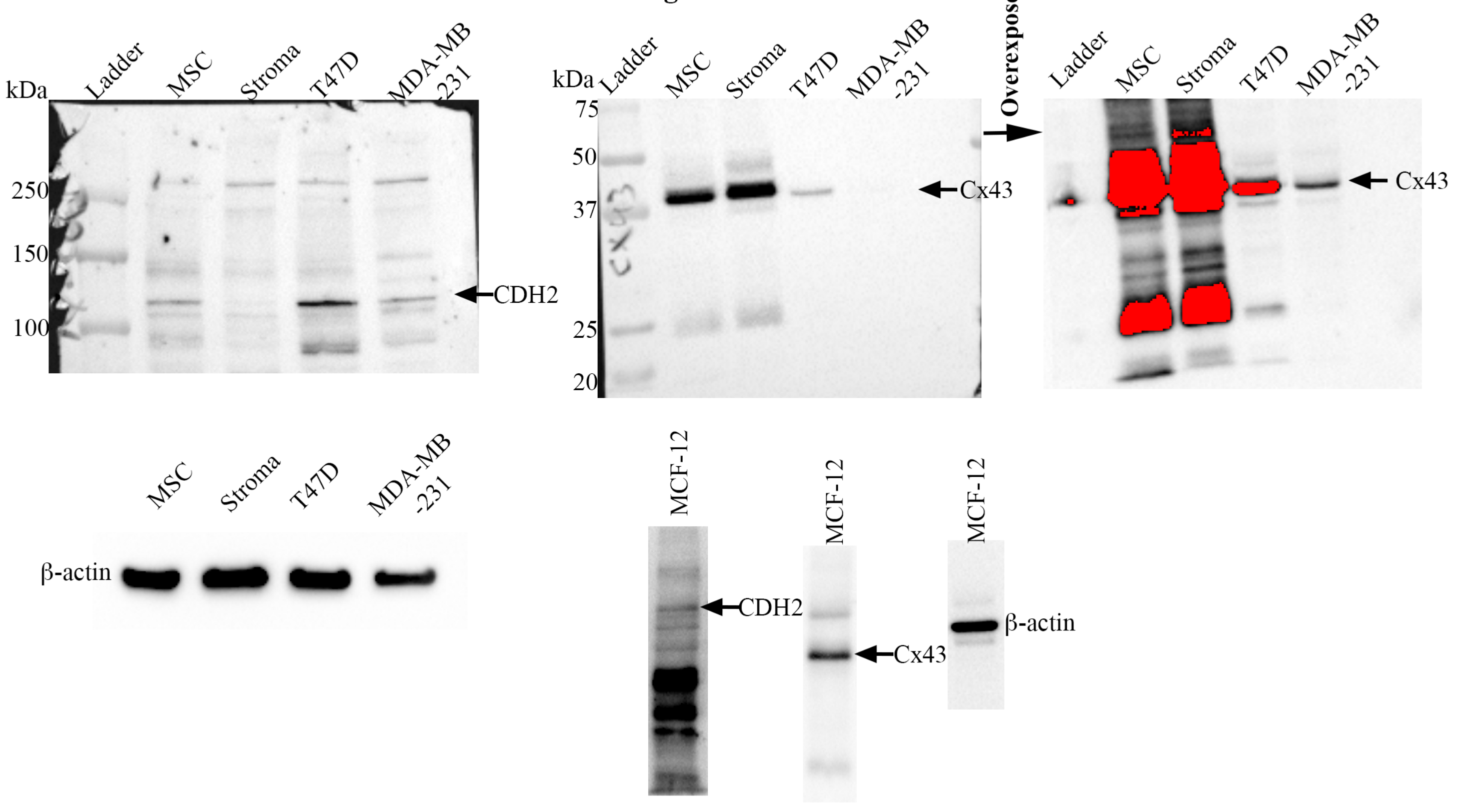

kDa
Ladder
MSC
Stroma
T47D
MDA-MB -231
250
150
100
CDH2
kDa
75
50
37
25
20
Cx43
Overexposed
Ladder
MSC
Stroma
T47D
MDA-MB -231
Cx43
MSC
Stroma
T47D
MDA-MB -231
β-actin
MCF-12
CDH2
MCF-12
Cx43
MCF-12
β-actin

Figure: 3E

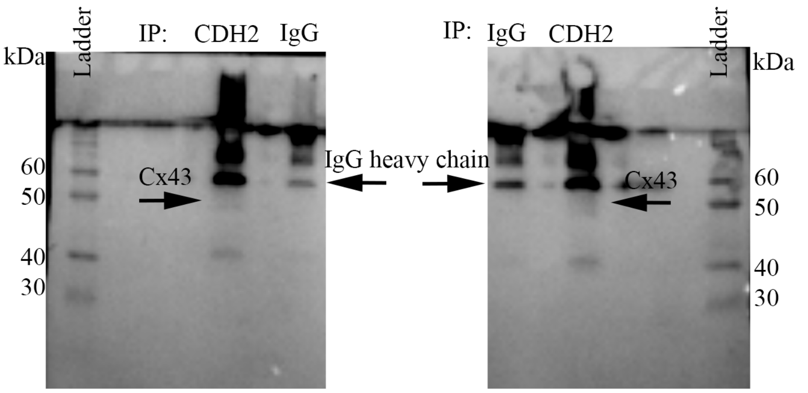

Figure: 3F

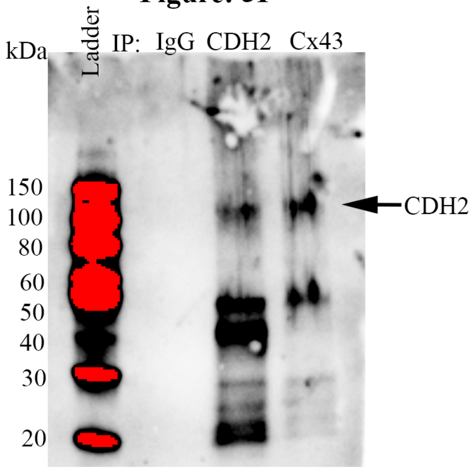

Figure 3G

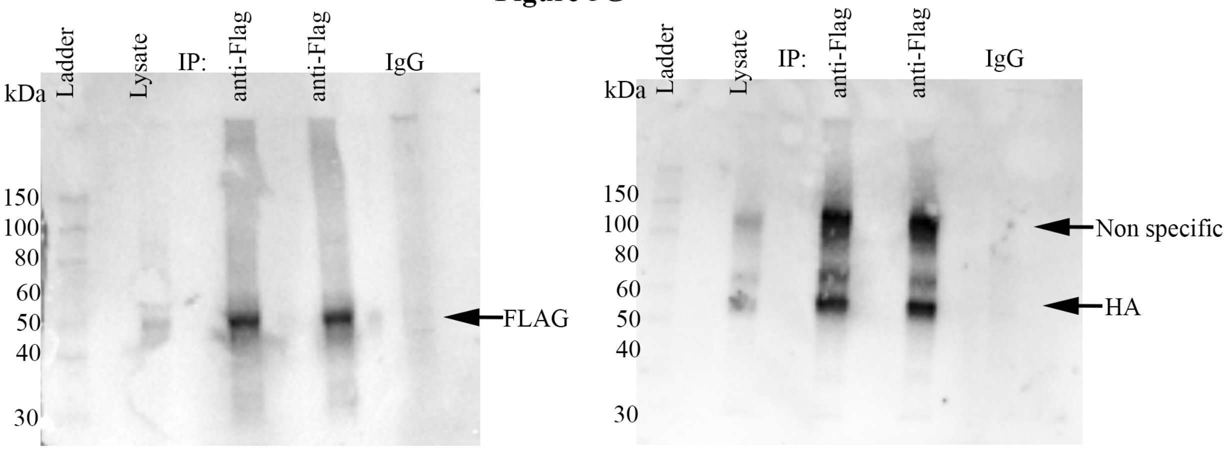

Figure 3H

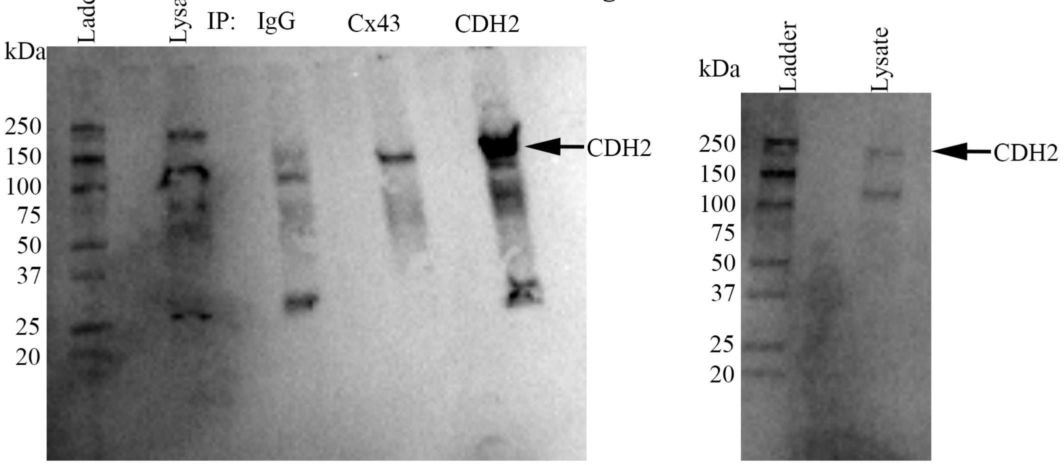

Figure 5G

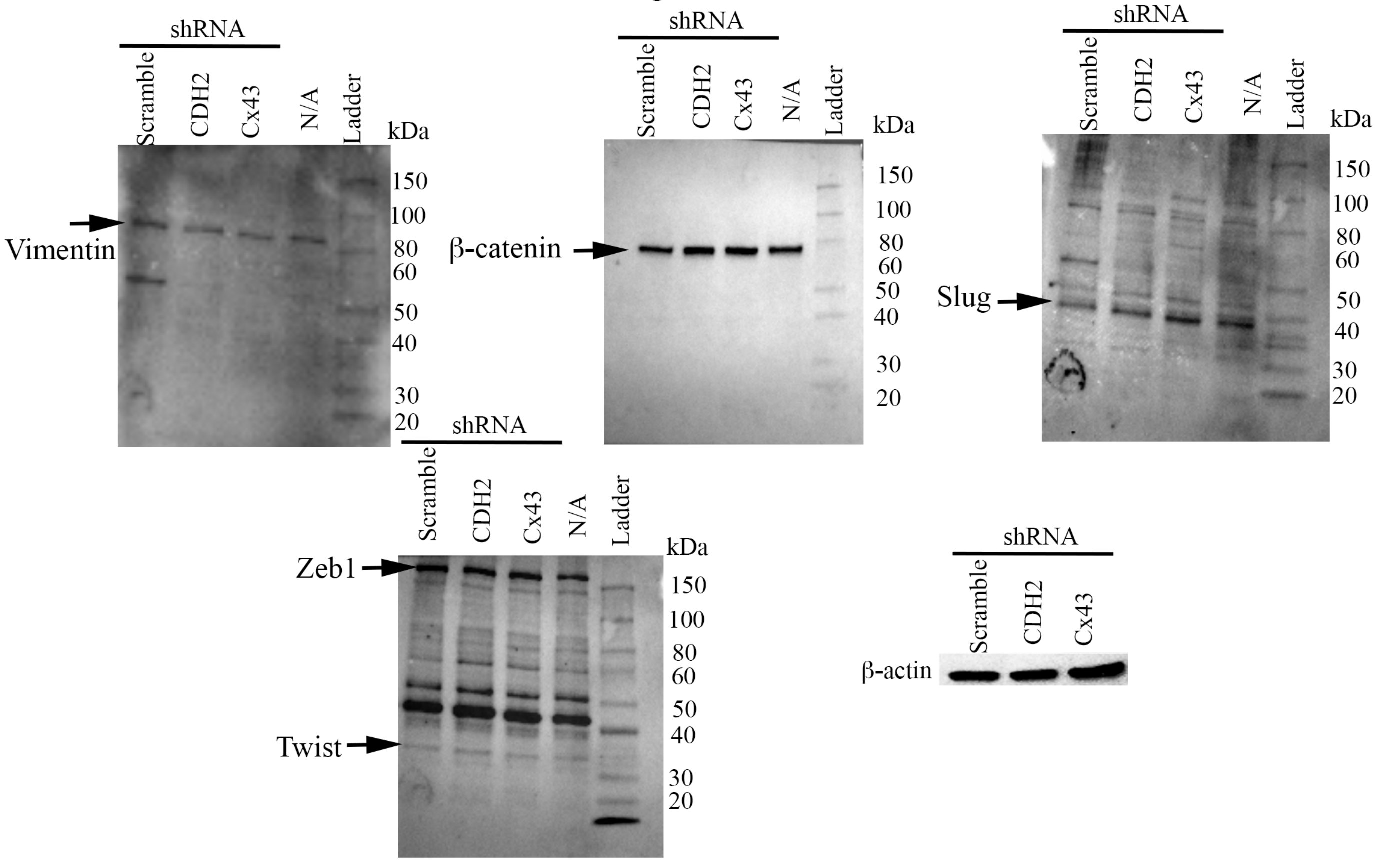

Figure 5G (second set)

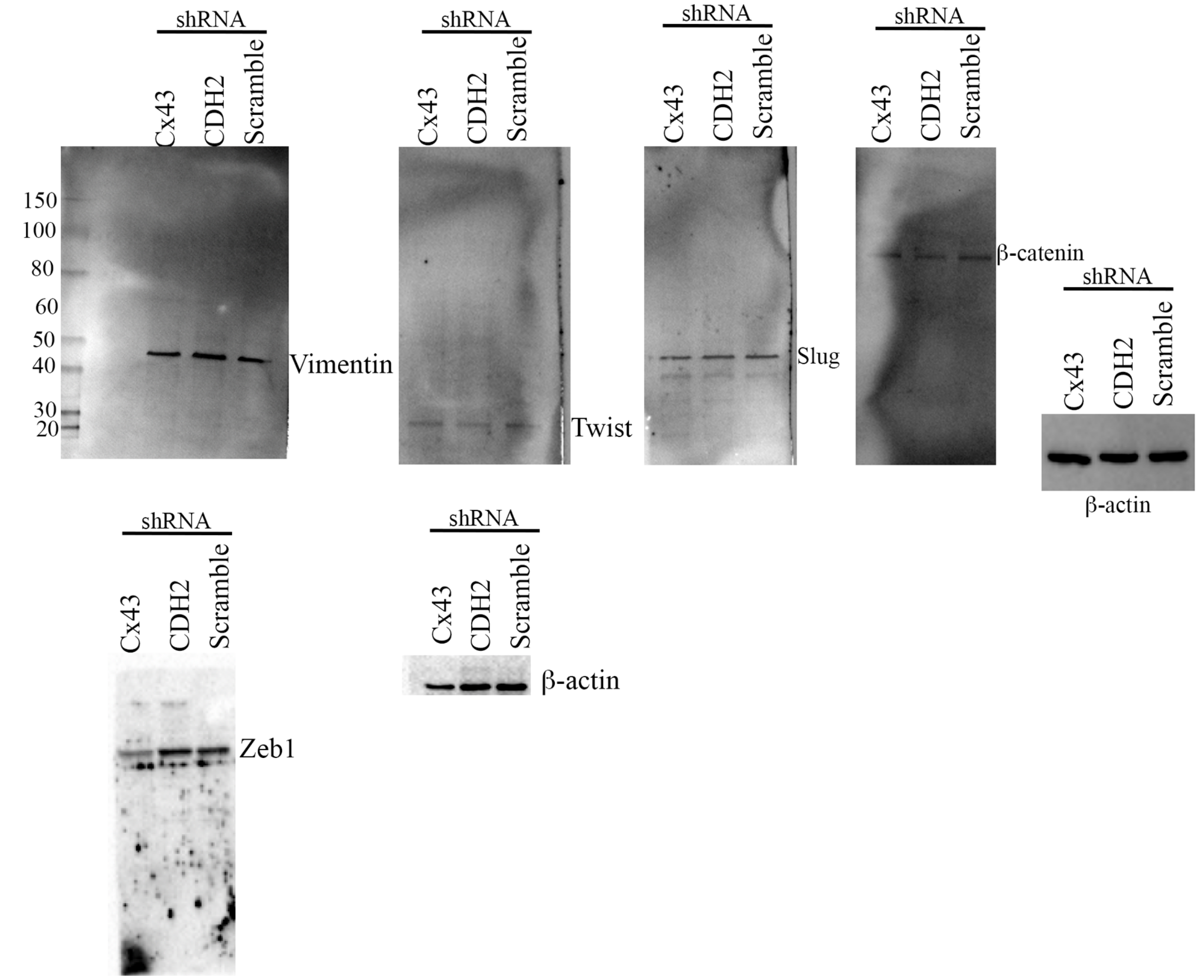

Figure 5H

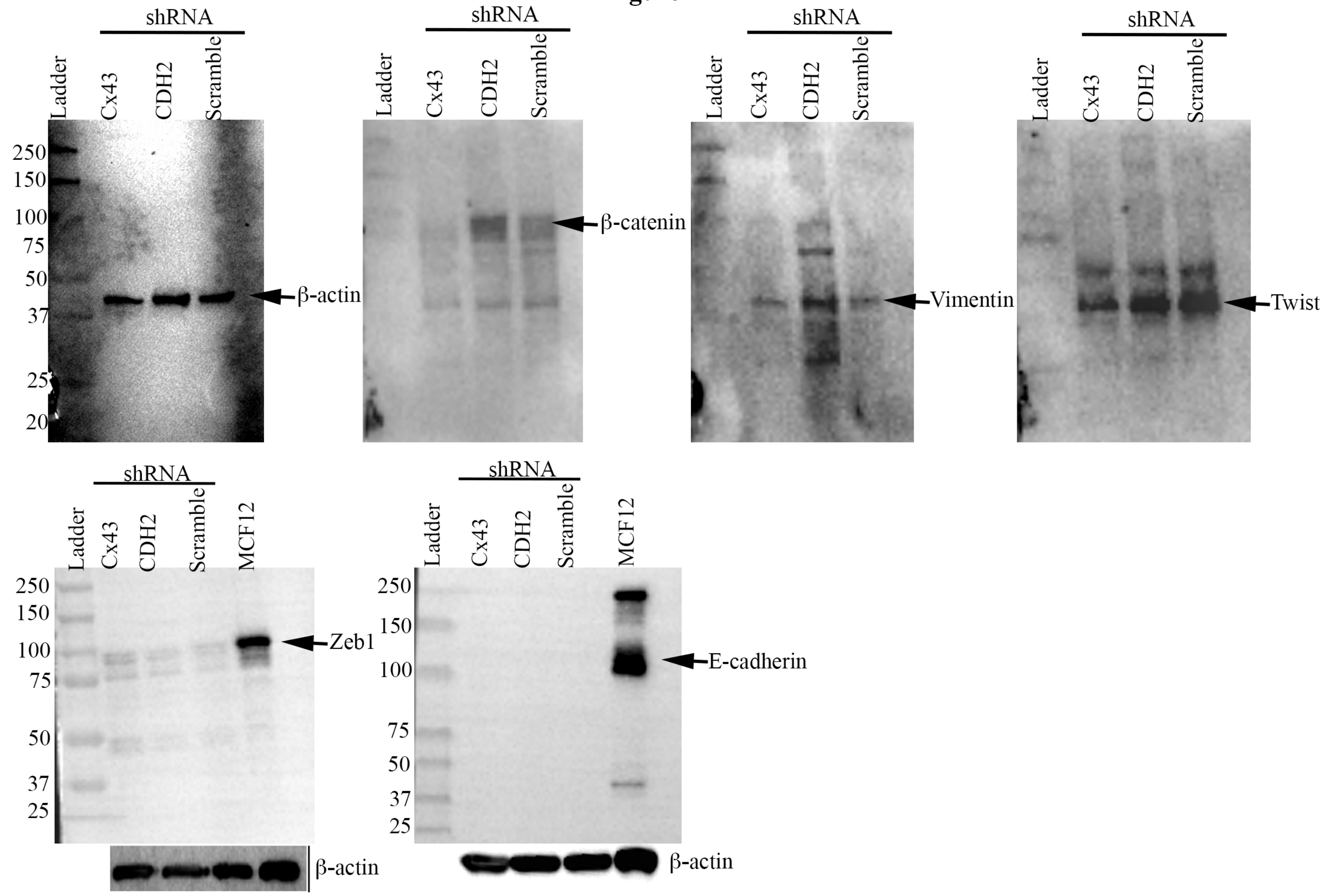
shRNA
Ladder
Cx43
CDH2
Scramble
250
150
100
75
50
37
25
20
β-actin
β-catenin
Vimentin
Twist
MCF12
Zeb1
E-cadherin

Figure 5I

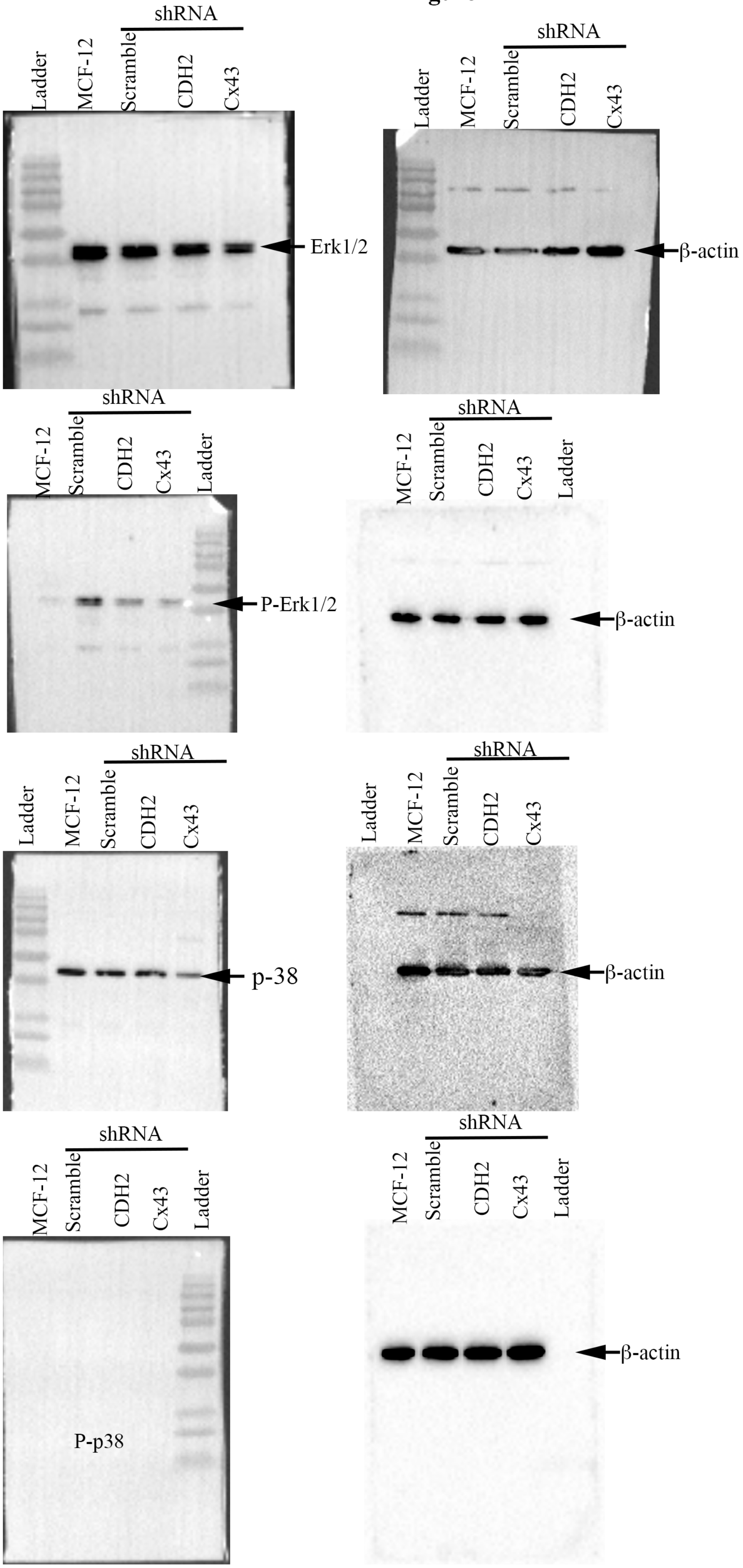

shRNA
Ladder
MCF-12
Scramble
CDH2
Cx43
Erk1/2
β-actin
P-Erk1/2
p-38
P-p38

Figure 6G

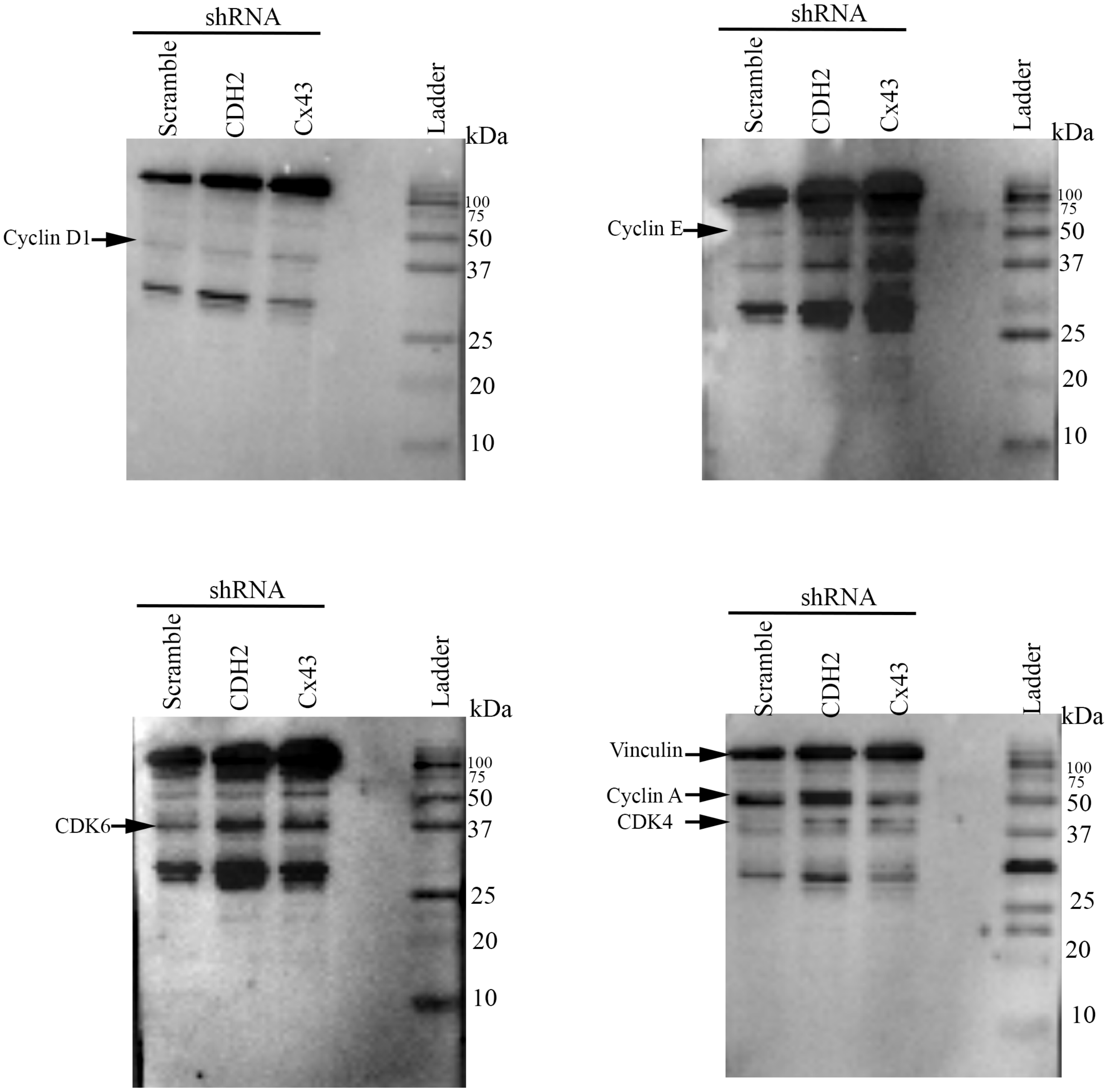
shRNA
Scramble
CDH2
Cx43
Ladder
kDa
Cyclin D1
100
75
50
37
25
20
10
shRNA
Scramble
CDH2
Cx43
Ladder
kDa
Cyclin E
100
75
50
37
25
20
10
shRNA
Scramble
CDH2
Cx43
Ladder
kDa
CDK6
100
75
50
37
25
20
10
shRNA
Scramble
CDH2
Cx43
Ladder
kDa
Vinculin
Cyclin A
CDK4
100
75
50
37
25
20
10

Figure 7B

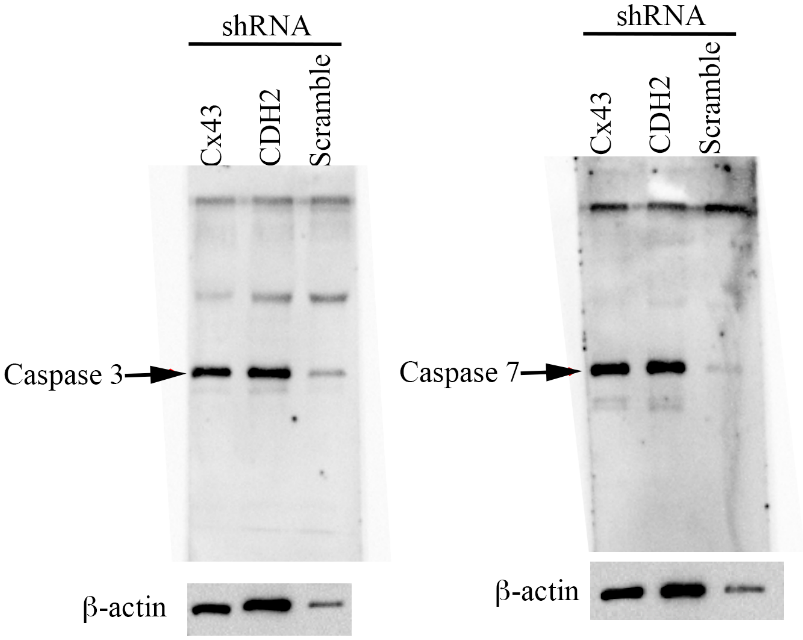

Figure 7D

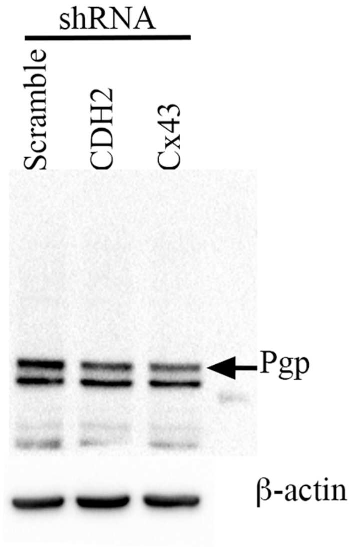

Figure 7B (2nd replicate)

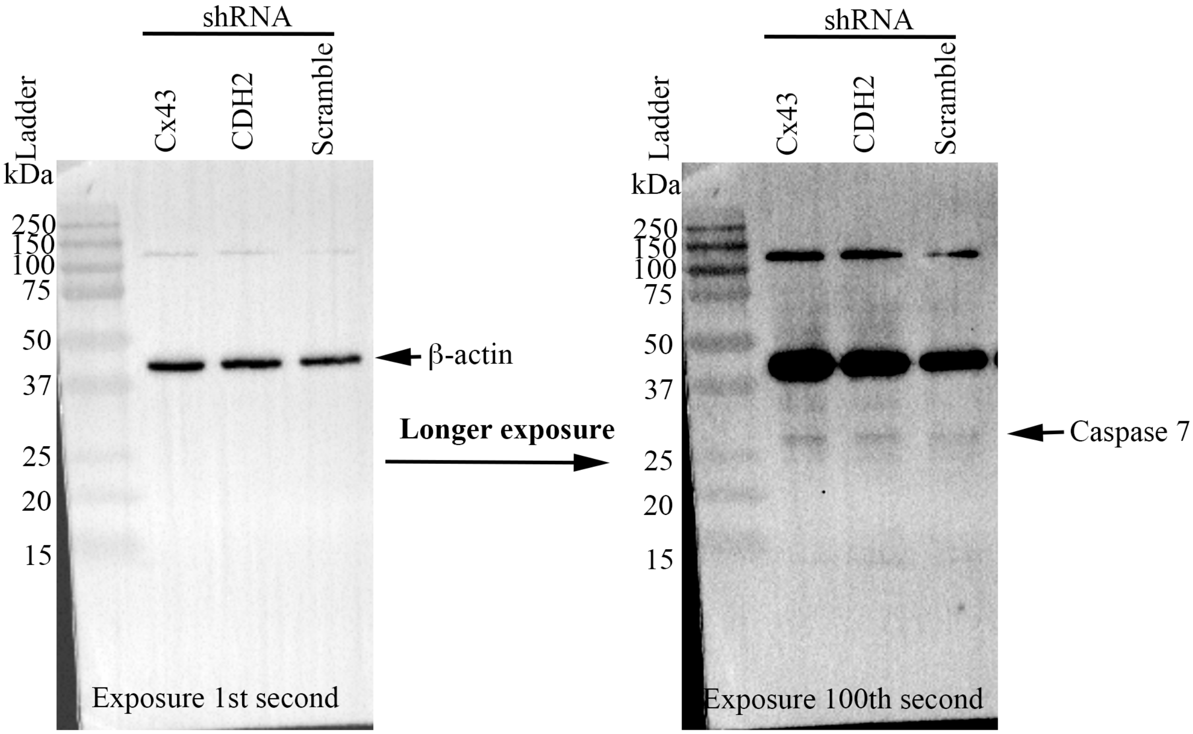

Figure S1D

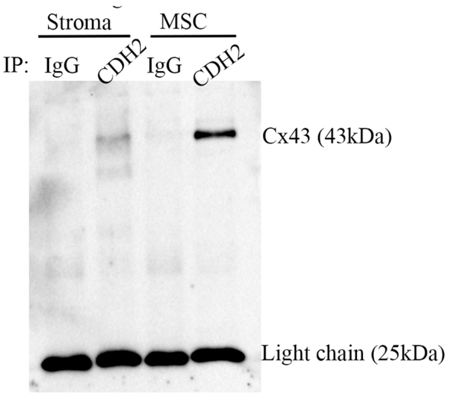

Figure S1F

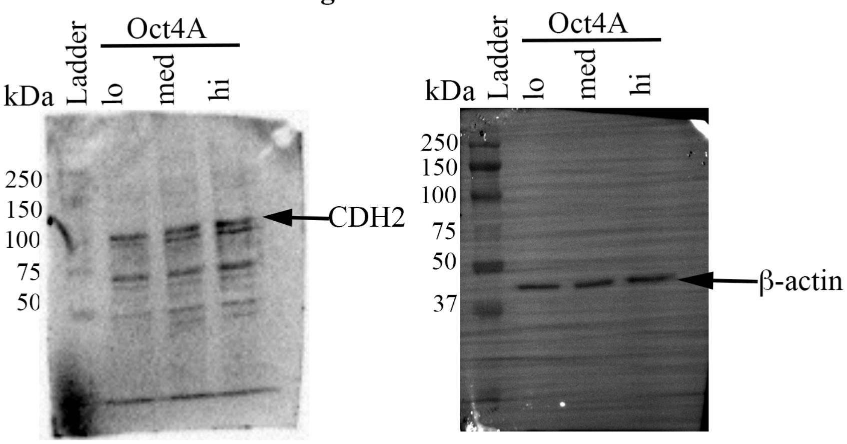

Figure S1H

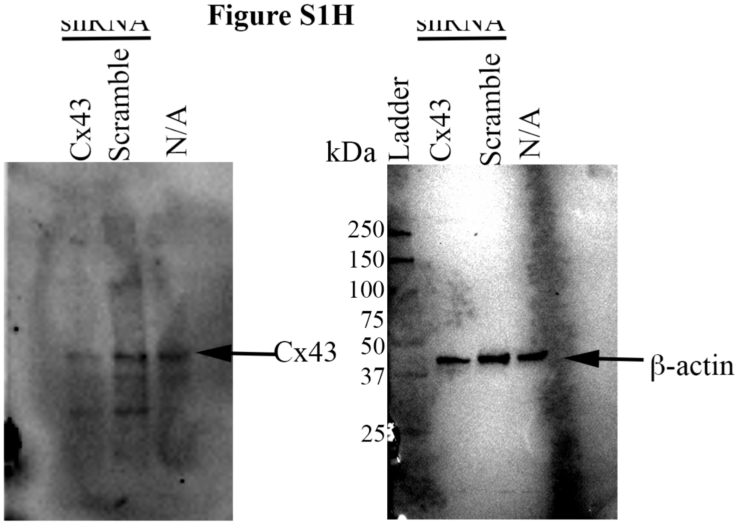

Figure S1K

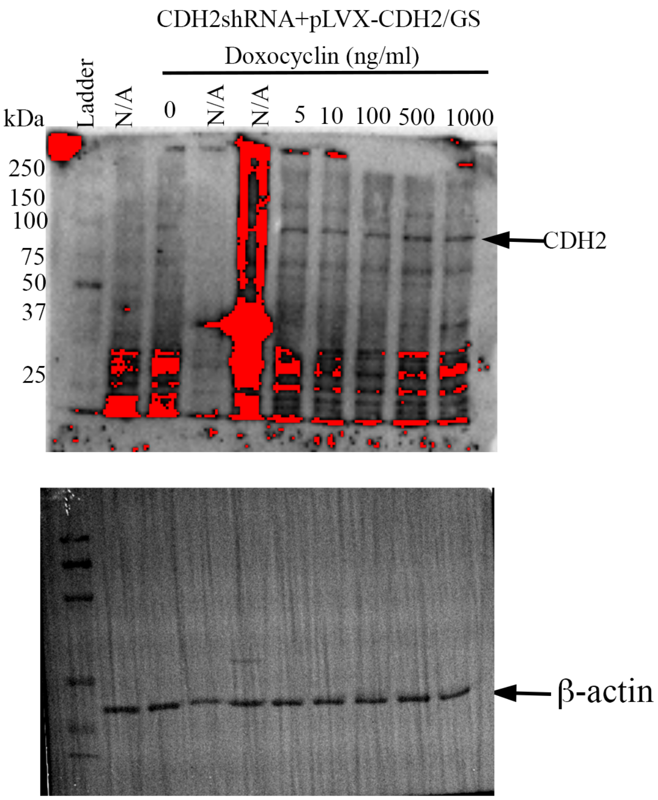

Figure S1L

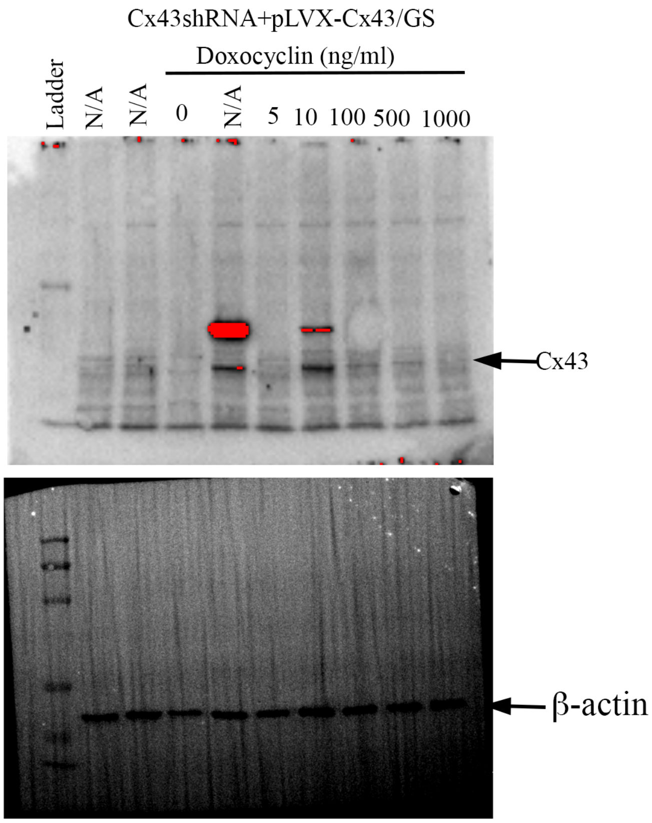

Supplement: Supplementary file 1 [file LSA-2020-00969_SdataF1.pdf]
